# Supplementary material for: In Vivo Analysis of Optic Fissure Fusion in Zebrafish: Pioneer Cells, Basal Lamina, Hyaloid Vessels, and How Fissure Fusion is Affected by BMP
Source: Int J Mol Sci. 2020 Apr 16;21(8):2760. doi: 10.3390/ijms21082760 (PMC7215994; doi:10.3390/ijms21082760)
Supplement: Supplementary file 1 [file ijms-21-02760-s001.zip › figure legends .pdf]

Figure S1:separated fluorophore channels of imaging data of Figure 1. Besides the arrows, as in Figure 1, asterisks have been added to A and A′ to enable easier comparison of domains and cells.

Figure S2: Combinations of the 3 channels of Figure 2 (D-G) from proximal to distal. (A-D) merge of laminin (yellow) and lyntdTomato (magenta) channel, (A´-D´) merge of laminin (yellow) and *tg(rx2:eGFPcaax)* (green) channel and (A´´-D´´) merge of lyntdTomato (magenta) and *tg(rx2:eGFPcaax)* (green). (A´´- H) Comparison of immuno stained embryos (A´´-D´´) versus in vivo imaging of embryos. *tg(rx2:eGFPcaax)* embryos where injected with lyntdTomato mRNA into the zygote and grown until 43hpf, they were in vivo imaged with a confocal microscope, fixed and immuno stained for laminin and GFP and imaged again. (The optic fissure depicted in A´´- D´´ is not from the same embryo as in E-H but they are siblings). Optical sections were taken with a z-spacing of 12µm (A´´-B´´) and 6 µm each (B´´-D´´). Optical sections for E-H were taken with a z-spacing of 9µm each. Hyaloid vein (asterisk´s). Scale bars 25µm, sagittal view, nasal to the left.

Figure S3: cross of *tg(Ncad:NcadGFP)* and *tg(hsp:bmp4)*, heat shocked at 26 hpf and imaged at approximately 38 hpf. In control and bmp induced embryos the apical domains marked by the Ncad-GFP are reaching into the optic fissure (arrows), to a comparable degree (*tg(hsp:bmp4)* n=9 , control n=4)Scale bar 25µm

Figure S4: distal z-projection of the control *tg(fli1a:eGFP)* embryo from Figure 7A-D, comparable to H, superficial endothelial cells were cropped from the images to enable the view on only dorsal (drv) and nasal radial vessel (nrv) as well as the annular vessel. Scale bar 50µm
