# Supplementary material for: In Vivo Analysis of Optic Fissure Fusion in Zebrafish: Pioneer Cells, Basal Lamina, Hyaloid Vessels, and How Fissure Fusion is Affected by BMP
Source: Int J Mol Sci. 2020 Apr 16;21(8):2760. doi: 10.3390/ijms21082760 (PMC7215994; doi:10.3390/ijms21082760)
Supplement: Supplementary file 1 [file ijms-21-02760-s001.zip › movie legends .pdf]

Supplemental Movie M1: Corresponding to Figure 1B-F. Zygotic microinjection of lyntdTomato mRNA (magenta), *tg(rx2:eGFPcaax)* transgenic reporter line (green). Onset of imaging: 37hpf, imaging temperature 28°C scale bar 25µm, lateral view, nasal to the left.
Supplemental Movie M2: Corresponding to Figure 3 A-D. Zygotic microinjection of lyntdTomato mRNA (magenta), *Ncad:Ncad-GFP* transgenic reporter line (green). Onset of imaging: 32hpf, imaging temperature 28°C, onset of movie 6 hrs after onset of imaging, scale bar 25µm, lateral view, nasal to the left.
Supplemental Movie M3: Corresponding to Figure 4A-C. Zygotic microinjection of lyntdTomato mRNA (magenta), *tg(fli1a:eGFP)* transgenic endothelial reporter (green). Onset of imaging: 28hpf, imaging temperature 28°C scale bar 25µm, lateral view, nasal to the left.
Supplemental Movie M4: Corresponding to Figure 4D-F. Zygotic microinjection of lyntdTomato mRNA (magenta), *tg(fli1a:eGFP)* transgenic endothelial reporter (green). Onset of imaging: 28hpf, imaging temperature 28°C scale bar 25µm, lateral view, nasal to the left.
Supplemental Movie M5: Corresponding to Figure 5C-F. Control embryo heat shocked at 26 hpf. Zygotic microinjection of lyntdTomato mRNA (magenta), *tg(rx2:eGFPcaax)* transgenic reporter line (green). Onset of imaging: 30hpf, imaging temperature 28°C scale bar 25µm, lateral view, nasal to the left.
Supplemental Movie M6: Corresponding to Figure 5G-J. *tg(hsp70l:bmp4)* embryo heat shocked at 26 hpf. Zygotic microinjection of lyntdTomato mRNA (magenta), *tg(rx2:eGFPcaax)* transgenic reporter line (green). Green fluorescence in the heart originates from the transgenesis marker *myl7:eGFP*. Onset of imaging: 30hpf, imaging temperature 28°C scale bar 25µm, lateral view, nasal to the left.
Supplemental Movie M7: Corresponding to Figure 5A-D. Control embryo heat shocked at 26 hpf. Zygotic microinjection of H2B-eGFP mRNA (green), *tg(sox10:lyntdTomato)* transgenic reporter line (magenta). Onset of imaging: 34hpf, imaging temperature 28°C scale bar 25µm, lateral view, nasal to the left.
Supplemental Movie M8: Corresponding to Figure 5E-H. *tg(hsp70l:bmp4)* embryo heat shocked at 26 hpf. Zygotic microinjection of H2B-eGFP mRNA (green), *tg(sox10:lyntdTomato)* transgenic reporter line (magenta). Green fluorescence in the heart originates from the transgenesis marker *myl7:eGFP*. Onset of imaging: 34hpf, imaging temperature 28°C scale bar 25µm, lateral view, nasal to the left.
Supplemental Movie M9: Corresponding to Figure 5I-L. Control embryo heat shocked at 26 hpf. Zygotic microinjection of lyntdTomato mRNA (magenta), *tg(fli1a:eGFP)* transgenic endothelial reporter (green). Onset of imaging: 32hpf, imaging temperature 28°C, onset of movie 5 hrs after onset of imaging 28°C scale bar 25µm, lateral view, nasal to the left.
Supplemental Movie M10: Corresponding to Figure M-P. *tg(hsp70l:bmp4)* embryo heat shocked at 26 hpf. Zygotic microinjection of lyntdTomato mRNA (magenta), *tg(fli1a:eGFP)* transgenic endothelial reporter (green). Onset of imaging: 32hpf, imaging temperature 28°C, onset of movie 5 hrs after onset of imaging 28°C scale bar 25µm, lateral view, nasal to the left.
