# Supplementary figures and images for: In Vivo Analysis of Optic Fissure Fusion in Zebrafish: Pioneer Cells, Basal Lamina, Hyaloid Vessels, and How Fissure Fusion is Affected by BMP
Source: Int J Mol Sci. 2020 Apr 16;21(8):2760. doi: 10.3390/ijms21082760 (PMC7215994; doi:10.3390/ijms21082760)

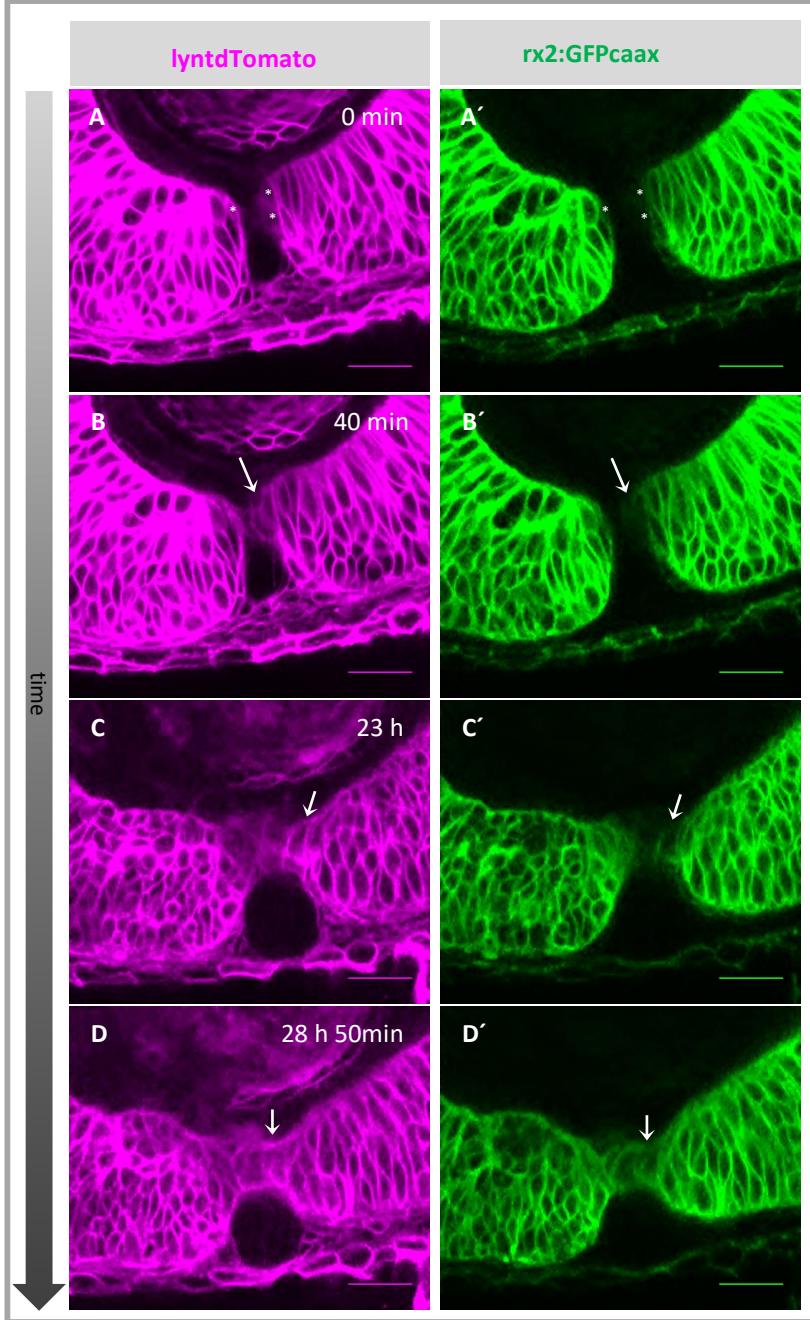

Revised Figure 1 Supplement Eckert et al., 2020

Supplement: Supplementary file 1 [file ijms-21-02760-s001.zip › figues/Figure 1 Supplement.pdf]

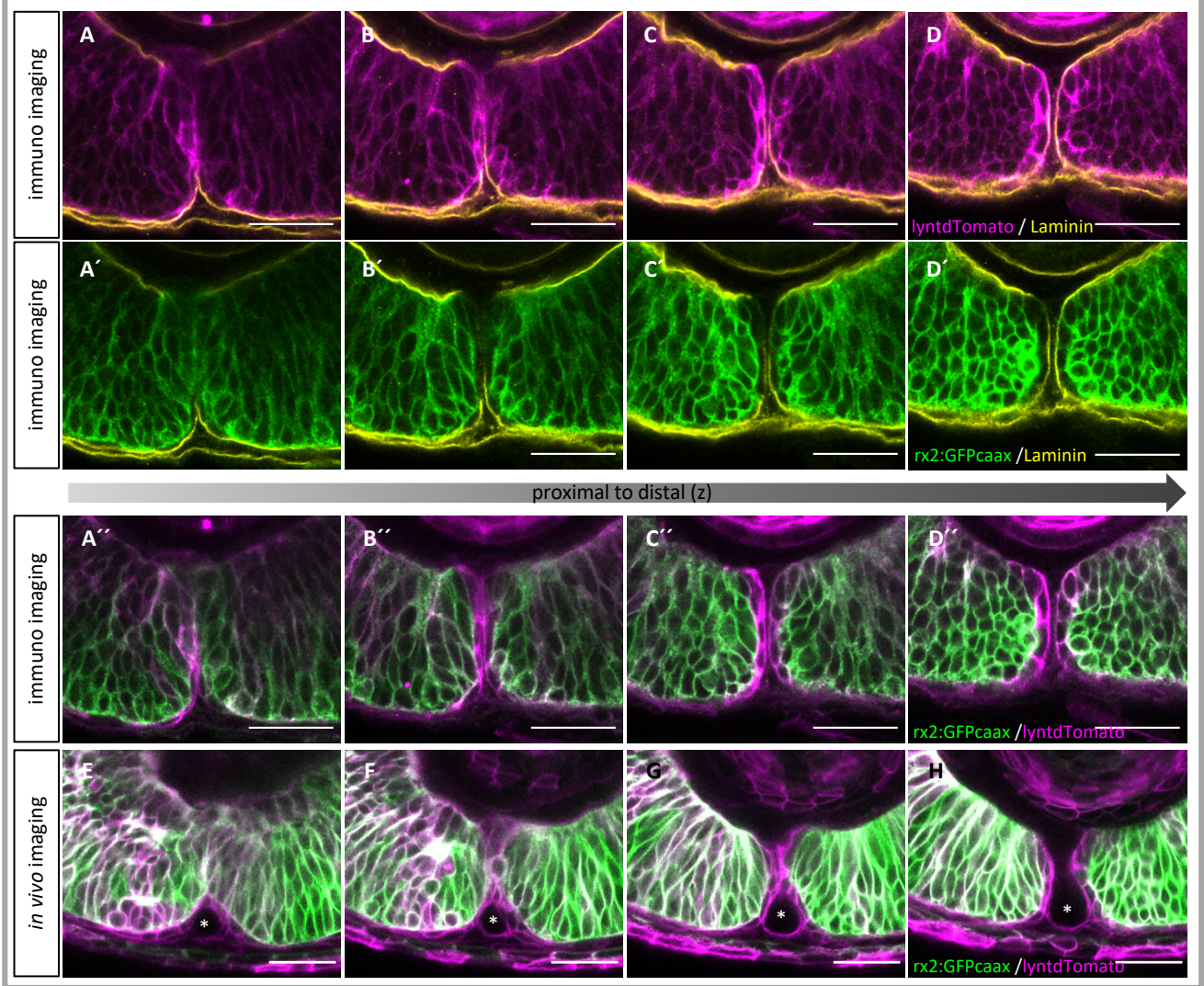

Figure 2 Supplement Eckert et al., 2020

Supplement: Supplementary file 1 [file ijms-21-02760-s001.zip › figues/Figure 2 Supplement.pdf]

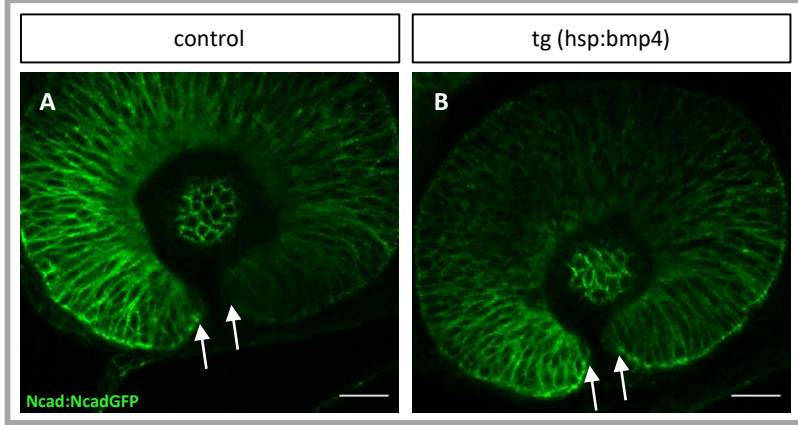

Figure 5 Supplement Eckert et al., 2020

Supplement: Supplementary file 1 [file ijms-21-02760-s001.zip › figues/Figure 3 Supplement.pdf]

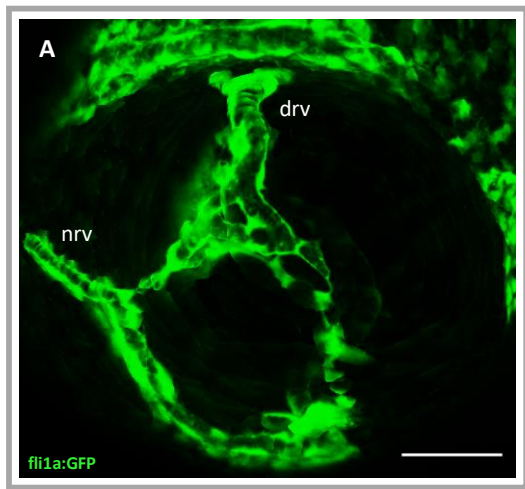

Figure 7 Supplement Eckert et al., 2020

Supplement: Supplementary file 1 [file ijms-21-02760-s001.zip › figues/Figure 4 Supplement.pdf]
